# From Joy to Generativity: 2-Year Shift in Hip-Hop Dance Teams Aged 65+ at a National Competition

**DOI:** 10.1093/geroni/igaf122.4352

**Published:** 2025-12-31

**Authors:** Atsuko Miyazaki

**Affiliations:** The University of Tokyo, Tokyo, Tokyo, Japan

## Abstract

Community-based hip-hop dance teams may do more than keep older adults moving—they may shift motives from enjoyment to contribution in later life. We examined two-year within-person change among older adults engaged in community hip-hop dance teams in Japan. At baseline (Year 1), 79 participants across seven municipalities were surveyed; longitudinal analyses included 62 participants (mean age 75.2±6.1; 96.8% women) from six municipalities with data at both Year 1 and Year 2. All teams practiced hip-hop and competed annually in a national competition. By Year 2, the mean participation duration was 2.73±0.99 years. A 40-item questionnaire spanned seven psychosocial domains (Helping Others, Belongingness, Relationships, Status, Social Identity, Enjoyment, Information). Change scores (Year 2 minus Year 1) were tested against zero using one-sample t-tests with Cohen’s d and 95% confidence intervals; participation averaged 2.16 sessions/week for 49.35 minutes/session. Prosocial orientation increased modestly: Helping Others Δ = +0.76 (95% CI 0.15–1.37; d≈0.32). Other domains showed small, directionally positive but non-significant changes—Status Δ = +0.63 (−0.29–1.55), Belongingness Δ = +1.47 (−0.40–3.34), Relationships Δ = +0.31 (−0.52–1.14), Information Δ = +0.19 (−0.49–0.87), Social Identity Δ = −0.02 (−1.12–1.08)—while Enjoyment declined slightly, Δ = −0.55 (−1.59–0.49). Participants frequently noted “more friends,” “moving more every day,” and “greater purpose,” echoing the quantitative pattern. Over two years, community hip-hop dance teams were associated with a small yet consistent shift from initial enjoyment toward contribution and connectedness. Although most effects were small and CIs included zero, the Helping Others CI did not, suggesting practical salience; implications include peer-mentor roles, structured helping opportunities, and intergenerational sessions.

